# A theoretical approach to zero-reflection toroidal curved metasurfaces

**DOI:** 10.1038/s41598-023-33896-4

**Published:** 2023-04-24

**Authors:** Hosein Allahverdizade, Sina Aghdasinia, Hemn Younesiraad, Mohammad Bemani

**Affiliations:** 1 Department of Electrical and Computer Engineering, University of Tabriz, Tabriz, 5166616471 Iran; 2 Department of Electrical Engineering, Faculty of Engineering, University of Kurdistan, Sanandaj, 6617715175 Iran

**Keywords:** Materials science, Electrical and electronic engineering

## Abstract

In this paper, we investigate the electromagnetic response of metasurfaces due to excitation of the toroidal moment. A toroidal curved metasurface analyzad using a novel theoretical solution based on the Fourier analysis to evaluate the localized fields. Analyzing localized near-field interactions are crucial in investigating the excited trapped modes and enables us to optimize the reflection properties of the proposed metasurface. Optimization is accomplished using graphene layer and resulted a hybrid dielectric-graphene structure with near-zero reflection properties.

## Introduction

Approaches for calculating scattered electromagnetic fields of metasurfaces have been proposed in the past, such as using periodic Green’s function (Floquet-Bloch) or the GSTC method^1^, but the fundamental issue with applying these methods is that the structures must have a minimum of periodicity in order to be employed and they are unsuitable for calculating near-field responses.

**Figure 1:**
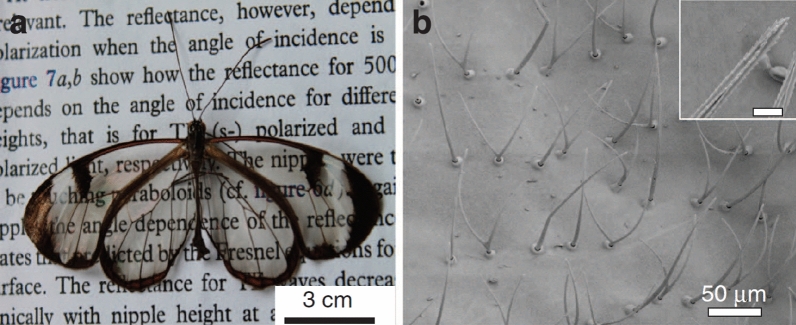
(**a**) Images of glasswing butterflies (Greta oto). (**b**) The image of the transparent area indicates that this area of the wing is covered in bristles or micro-hairs that are in nano scale^4^.

A zero-reflection structure is required to achieve transparency. This characteristic is present in the wings of the remarkable species of butterfly known as Greta oto Fig. 1a^2,3^. According to a recent study^3^, Greta Otto’s wings have asymmetrically dispersed nanostructures in the transparent areas Fig. 1b. It’s interesting that this aspect of the butterfly’s wings is what makes it transparent from different angles^4^. This observation motivates us to investigate analytical approaches of non-periodic nano-structures.

The electromagnetic response of metasurfaces can be related to traditional electric and magnetic dipoles or their complex combinations known as multipoles^5^. A toroidal dipole is the third member of localized electromagnetic excitations which is created by a current circulating on the surface of a torus and has been observed in solid-state physics^6^. The toroidal response of metasurfaces was experimentally observed in microwave regime^7^ and then theoretically scaled to the THz regime^8^. The performances of these metasurfaces are usually limited by radiative and nonradiative losses where nonradiative losses can by reduced by employing materials of low loss such as dielectric^9,10^ likewise the solution employed in nontoroidal metasurfaces^11^.

Radiative losses can be regulated by designing the asymmetric all-dielectric unit cells to excite a high-quality factor resonant response known as Fano resonance in which the radiative damping can be efficiently suppressed by trapped mode and leads to the reduction of radiative losses^12^. Trapped modes that excited in such asymmetrical structures are included in the the concept of bound states in the continuum (BIC)^13^. A strong link between the toroidal dipole resonance and the BIC was defined in the context of all-dielectric metasurfaces^14^. To construct a trapped mode supporting metasurface, we utilize a set of identical subwavelength unit cells with two asymmetric high-refractive index Silicon particles made in the form of modified nanodisks.

Trapped modes lead to a strong near-field enhancement where the located electromagnetic fields becomes notable. We utilize a novel theoretical approach to analytically evaluate localized electromagnetic fields near the all-dielectric unit cells of trapped mode supporting metasurface.

**Figure 2:**
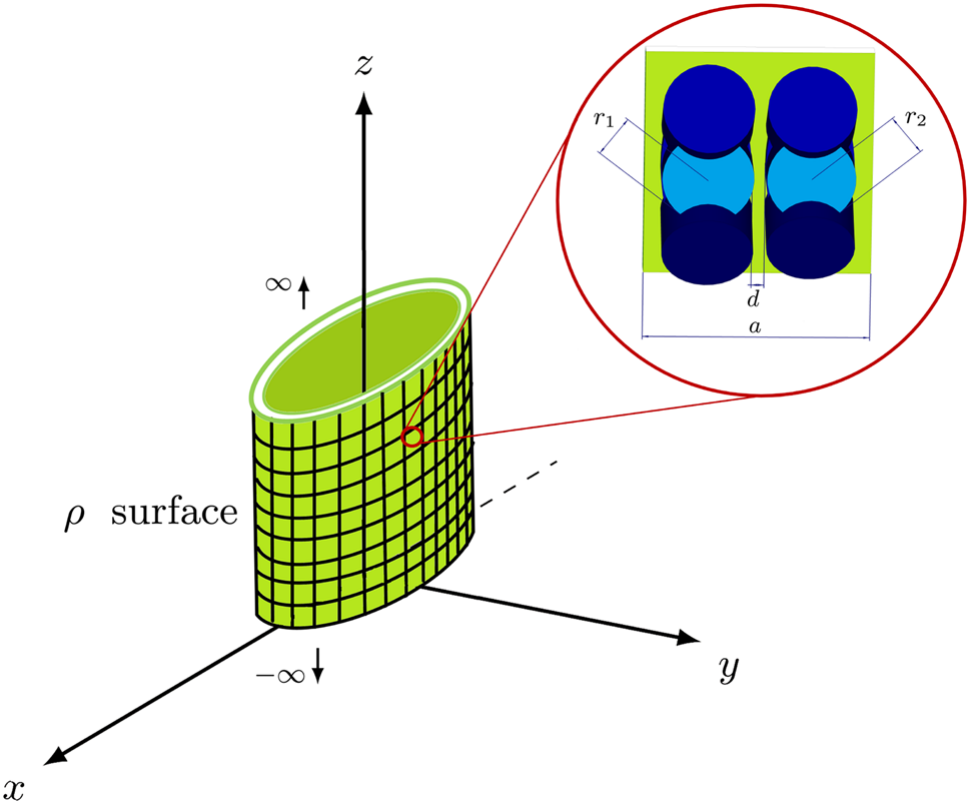
The sampled curved $\rho$ surface and top view of each modified unit cell with $r_1=16$ $\upmu$m, $r_2=15.5$ $\upmu$m, $d=3.38$ $\upmu$m and $a=80$ $\upmu$m.

This evaluation provides the capability of manipulating the reflection coefficient for the surface. Our investigations should support every possible surface shape in the practical applications of such metasurfaces as sensing^15,16^. To accomplish a comprehensive solution, we consider a cylindrical surface where any arbitrary surface can be fitted on a cylindrical surface with a defined cross-section.

Since all-dielectric curved metasurfaces are composed of multi subwavelength cells, their optical properties are related to resonant features of each constitutive unit cell resonator and its mutual interaction with the other cells, trapped mode excitation does not merely satisfy the high-Q and zero-reflection properties which necessitates computing the localized electromagnetic fields to optimize such curved multi-cell metasurfaces.

The sampled curved metasurface was selected as $\rho$ surface in Fig. 2 with complete $\varphi$ angle which enables us to analyze the localized electromagnetic fields with Fourier series analysis in $2\pi$ interval which is discreted to 20 individual values to easily manipulate the sections to reduce the overall reflectivity.

Graphene is selected to optimize the obtained reflectivity from evaluated electromagnetic fields. Optimization can be implemented by varying the graphene chemical potential when the graphene layer is integrated with the proposed dielectric structure.

## Results and methods

### Theoretical formulation

The toroidal moment ${\textbf{T}}$ emerges from the multipole expansion of an arbitrary localized current distribution^6^. ${\textbf{T}}$ is defined in terms of current density distribution $\mathbf {j(r)}$, as follows

1 $$\begin{aligned} {\textbf{T}} = \frac{1}{{10c}}\int {\left[ { {{\textbf{r}}}( {{\textbf{r}}}.{\textbf{j}}) - 2{r^2}{\textbf{j}}} \right] {d^3}r} \end{aligned}$$

where *c* indicates the speed of light in free space. Equation (1) is satisfied by the following form of current density distribution

2 $$\begin{aligned} \mathbf {j(r)} = c \nabla \times \nabla \times \delta ( {{\textbf{r}}}) {\textbf{T}}. \end{aligned}$$

By decomposing current vector into longitudinal $\mathbf {j_\parallel }$ and transversal $\mathbf {j_\bot }$ parts, Eq. (1) can be recast into a more convenient form. $\mathbf {j_\parallel }$ does not contribute to the toroidal moment ${\textbf{T}}$^6^ but $\mathbf {j_\bot }$ can be written as the curl of the magnetization density $\mathbf {j_\bot }=c\nabla \times \mu ({\textbf{r}})$ and gives the toroidal moment in terms of magnetization density^17^

3 $$\begin{aligned} {\textbf{T}} = \frac{1}{2}\int {{{\textbf{r}}} \times \mu ({{\textbf{r}}}){d^3}r}. \end{aligned}$$

Equation (3) can be used to evaluate the toroidal moment for a finite system with known magnetization density. Representing the magnetization density by a distribution of localized magnetic moments $m_\alpha$ at sites $r_\alpha$ leads to the following equation for localized magnetization density $\mu _{\text {loc}}$

4 $$\begin{aligned} \mu _{\text {loc}} = \sum _{\alpha } {m_\alpha \delta ({{\textbf{r}}} - {{\textbf{r}}}_\alpha )} \end{aligned}$$

hence the toroidal moment takes the following form simplified to localized magnetic moments

5 $$\begin{aligned} {\textbf{T}} =\frac{1}{2}\sum _{\alpha } {{{{\textbf{r}}}_\alpha } \times {m_\alpha }}. \end{aligned}$$

**Figure 3:**
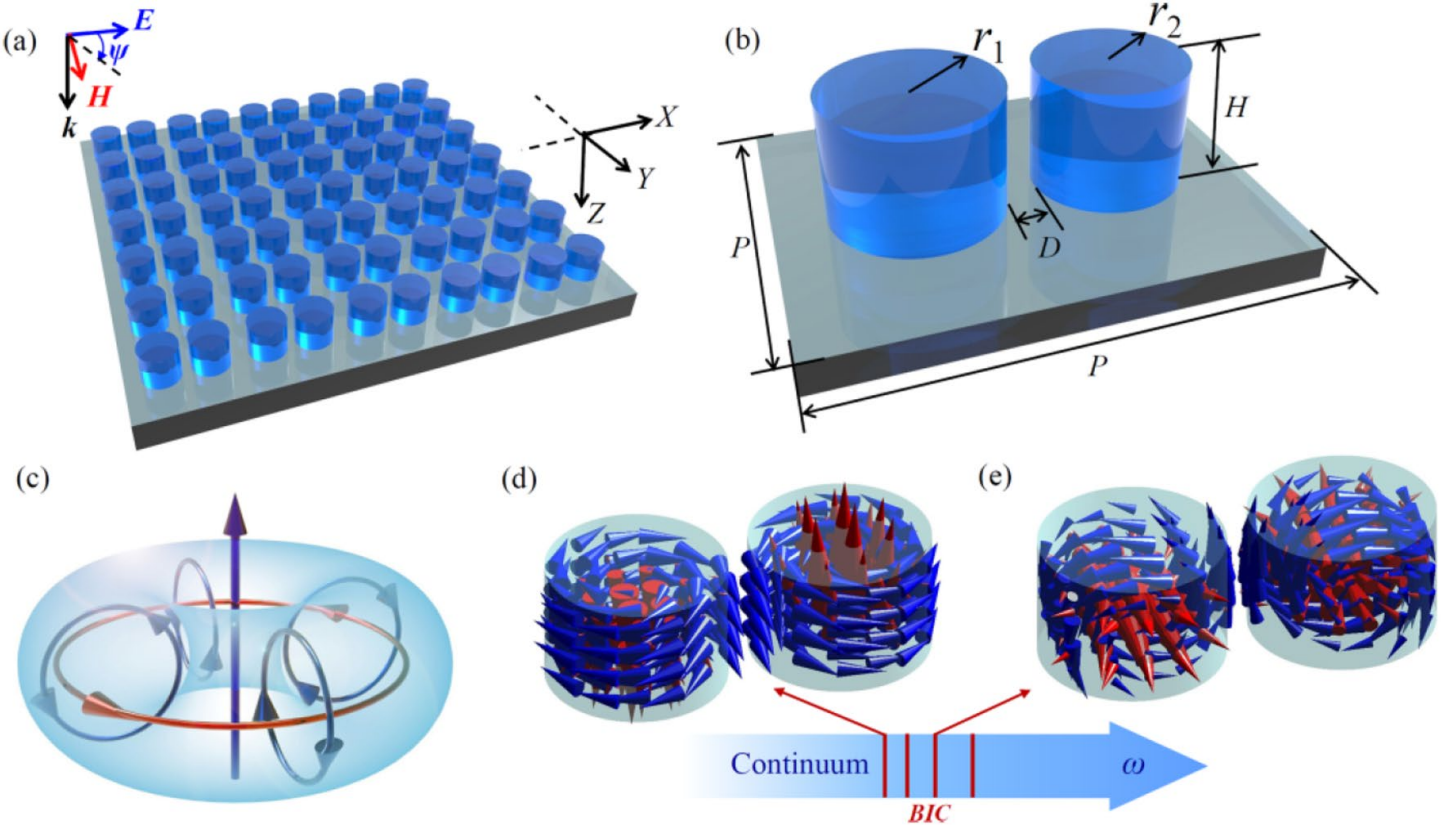
(**a**) A diagram of an all-dielectric metasurface. (**b**) The metasurface’s unit cell. $r_1,_2$ denotes the radius of the constituent nanodisks, *H* is the height of the disks, *D* is the distance between two nanodisks in the unit cell, and *P* is the lattice constant. (**c**) Diagram of a TD moment. (**d**) and (**e**) Two metasurface eigenmodes having TD moments along the *Y* and *Z* axes^18^.

Figure 3 shows a toroidal construction made up of two cylinders of various sizes. Based on Eq. (5), this structure clearly illustrates how to distribute magnetic moments in two opposite directions and so form a toroidal moment. Because of the simplicity of modeling and the ability of application to different forms figure, we will utilize this structure in our investigations^18^.

Respectively the potential caused by the toroidal moment $\mathbf {A^T}$ takes the following form due to the similarity of ${\textbf{T}}$ and the electrical moment ${\textbf{P}}$^19^

6 $$\begin{aligned} \mathbf {A^T} = j\frac{{{\textbf{T}}{e^{jk\left| {{{\textbf{r}}} - {\mathbf {r'}}} \right| }}}}{{4\pi \left| {{{\textbf{r}}} - {\mathbf {r'}}} \right| }}. \end{aligned}$$

The localized magnetic field at arbitrary sites, ${\textbf{H}}^{\text {loc}}$ is equal to the field caused by an external source, ${\textbf{H}}^\text {ext}$ and integral of the indicated potential caused by toroidal moment

7 $$\begin{aligned} {\textbf{H}}^\text {loc} = {\textbf{H}}^{\text {ext}} + \int {\nabla ' \times \mathbf {A^T}d\Omega '} \end{aligned}$$

however the toroidal moment is itself affected by external source. We know that $\frac{{{e^{jk\left| {{{\textbf{r}}} - {\mathbf {r'}}} \right| }}}}{{4\pi \left| {{{\textbf{r}}} - {\mathbf {r'}}} \right| }}$ term in Eq. (6) is the free-space Green function in the spherical coordinates^20^ which can be replaced with its equivalent in the cylindrical coordinates

8 $$\begin{aligned} \mathbf {A^T} = {\textbf{T}}G= {\textbf{T}}\frac{{1}}{4j}H_0^2(k\left| {\mathbf {\rho '}- \mathbf {\rho } } \right| ) \end{aligned}$$

where $H_0^2(\rho )$ is Hankel function of the second kind, zeroth order and by assuming ${{\textbf{H}}^{\text {ext}}}$ in the cylindrical coordinates, the total localized magnetic field can be defined in cylindrical coordinates which enables us to analyze surfaces like $\rho$ surface in Fig. 1 where $\rho$ is dependent to $\varphi$ angle.

**Figure 4:**
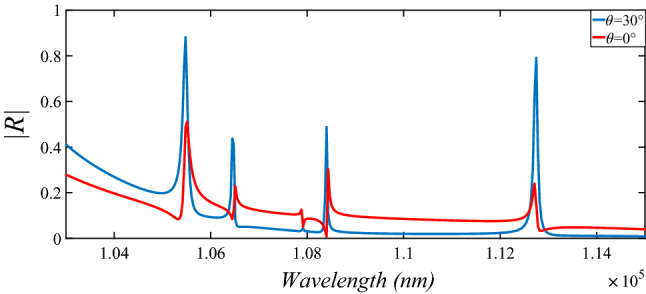
Overall reflection through wavelength for modified unit cell.

The toroidal moment can be defined on each unit cell of $\rho$ surface produced by non-directional magnetic moments of two nanodisks with *d* spacing

9 $$\begin{aligned} {\textbf{T}} = \frac{d}{2} {\hat{a}}_{\varphi '} \times \alpha _m {\textbf{H}}^\text {loc} \end{aligned}$$

due to $2\pi$ period of $\rho$ surface, ${\textbf{H}}^\text {loc}$ can be expanded in the Fourier series with $H_{l'}^{\text {loc},x'}$, $H_{l'}^{\text {loc},y'}$ and $H_{l'}^{\text {loc},z'}$ coefficients as follows

10 $$\begin{aligned} {\textbf{H}}^\text {loc}= \sum \limits _{l'} \left[ H_{l'}^{\text {loc},x'}{{\hat{a}}_x }+H_{l'}^{\text {loc},y'}{{\hat{a}}_y}+H_{l'}^{\text {loc},z'}{\hat{a}}_z \right] {e^{jl'\varphi '}} \end{aligned}$$

evaluating the toroidal potential caused by this expanded localized H-field, leads to calculating overall ${\textbf{H}}^\text {loc}$ in Eq. (7) by a novel approach in Methods. Another double Fourier series with $H_{l,l'}^{\text {s},x}$, $H_{l,l'}^{\text {s},y}$ and $H_{l,l'}^{\text {s},z}$ coefficients appears in calculated ${\textbf{H}}^\text {loc}$ as follows

11 $$\begin{aligned} {\textbf{H}}^\text {loc} = {\textbf{H}}^\text {ext} + \frac{d}{8}{\alpha _m}\sum \limits _l \Bigg [ \sum \limits _{l'} l' \bigg [H_{l,l'}^{\text {s},x}\hat{a_x}+H_{l,l'}^{\text {s},y}\hat{a_y} +H_{l,l'}^{\text {s},z}\hat{a_z} \bigg ] \Bigg ] e^{-jl\varphi } \end{aligned}$$

where $\alpha _m$ produced as we used a linear relationship between the magnetic field and the resulting magnetization which can be obtained in structures similar to a cylinder with *L* length and $2r_1$ diameter as^21^

12a $$\begin{aligned} \alpha _m= & {} \frac{1}{({n_s^2} - 1)g_m + 1} \end{aligned}$$

12b $$\begin{aligned} g_m= & {} ({S^2} - 1)\left[ {\frac{1}{2}S \ln \left(\frac{{S+1}}{{S-1}}\right)-1} \right] \end{aligned}$$

12c $$\begin{aligned} S= & {} \left[ 1 -\left( \frac{L}{2r_1} \right)^2 \right] ^{ - \frac{1}{2}} \end{aligned}$$

where $n_s=3.88$ is the index of refraction of the silicon nanodisk medium^22^. However our medium has been modified by embedding two extra $30^{\circ }$ inclined nonodisks with respect to main nanodisk as Fig. 1 to make the response stable for oblique incidents which is compared for 2 different external incidents in Fig. 4.

### Methods

This section describes how to compute a localized magnetic field using the previously mentioned potential formula (8). Since the localized magnetic field has a Fourier series form because of the $2\pi$ periodicity in our structure, we can use some basic Fourier series properties to solve the integral equation. Replacing Hankel part of *G* by double Fourier series with ${a}_{l,l'}$ coefficient and substituting Eq. (9) in (8), overall localized H-field equals

13 $$\begin{aligned} {\textbf{H}}^\text {loc} = {\textbf{H}}^\text {ext} + \frac{d}{2} {\alpha _m} \int {\nabla ' \times \bigg [ ({\hat{a}}_{\varphi '} \times {\textbf{H}}^\text {loc} ) \times \frac{1}{4j} \sum \limits _l \sum \limits _{l'} \left[ {a}_{l,l'}{e^{j(l'\varphi ' - l \varphi )}} \right] \bigg ] d\Omega '} \end{aligned}$$

where (${{\hat{a}}_{\varphi '}} \times {\textbf{H}}^\text {loc}$) can be calculated as

14 $$\begin{aligned} {{\hat{a}}_{\varphi '}} \times {\textbf{H}}^\text {loc}= \sum \limits _{l'} \left[ H_{l'}^{loc,z'} \cos \varphi '{\hat{a}}_x+ H_{l'}^{loc,z'}\sin \varphi '{\hat{a}}_y -(H_{l'}^{loc,x'}\cos \varphi '+H_{l'}^{loc,y'}\sin \varphi '){{\hat{a}}_z} \right] {e^{jl'\varphi '}} \end{aligned}$$

and results with respect to Fourier properties

15 $$\begin{aligned} {{\hat{a}}_{\varphi '}} \times {\textbf{H}}^\text {loc}= & {} \sum \limits _{{l'}} \Bigg [{\frac{H_{l'-1}^{loc,z'} + H_{l'+ 1}^{loc,z'}}{2} {\hat{a}}_x} + { \frac{H_{l'+1}^{loc,z'} - H_{l'- 1}^{loc,z'}}{2j} {\hat{a}}_y}-\left({\frac{H_{l'+1}^{loc,x'} + H_{l'-1}^{loc,x'}}{2}+\frac{H_{l'+1}^{loc,y'} - H_{l'-1}^{loc,y'}}{2j}}\right){\hat{a}}_z \Bigg ]e^{jl'\varphi '}\nonumber \\= & {} \sum \limits _{l'} \left[ b_{l'}^{x'}{\hat{a}}_x + b_{l'}^{y'}{\hat{a}}_y + b_{l'}^{z'} {\hat{a}}_z \right] e^{jl'\varphi '} \end{aligned}$$

where a simplified Fourier series expansion of (${{\hat{a}}_{\varphi '}} \times {\textbf{H}}^\text {loc}$) results with $b_{l'}^{x'}$, $b_{l'}^{y'}$ and $b_{l'}^{z'}$ coefficients. Replacing the determined (${{\hat{a}}_{\varphi '}} \times {\textbf{H}}^\text {loc}$) in (13) results

16 $$\begin{aligned} {\textbf{H}}^\text {loc} - {\textbf{H}}^\text {ext} = \frac{d}{8j}\alpha _m \int \nabla ' \times \Bigg [ \sum \limits _{l'} \left[ b_{l'}^{x'}{\hat{a}}_x + b_{l'}^{y'}{\hat{a}}_y + b_{l'}^{z'} {\hat{a}}_z \right] e^{jl'\varphi '} \times \sum \limits _l \sum \limits _{l'} \left[ {{{a}_{l,l'}}{e^{(jl'\varphi ' - jl\varphi )}}} \right] \Bigg ] d\Omega ' \end{aligned}$$

Based on the Fourier series properties, multiplication of two periodic terms in (16) results in an integrated double Fourier series in which the coefficients are the convulsions of $a_{l,l'}$ with $b_{l'}$s

17 $$\begin{aligned} {\textbf{H}}^\text {loc} - {\textbf{H}}^\text {ext} =\frac{d}{8j}\alpha _m \int \nabla ' \times \Bigg [ \sum \limits _l\bigg [\sum \limits _{l'} (b_{l'}^{x'}*a_{l,l'}) e^{jl'\varphi '}{\hat{a}}_x+(b_{l'}^{y'}*a_{l,l'})e^{jl'\varphi '}{\hat{a}}_y +(b_{l'}^{z'}*{a_{l,l'}})e^{jl'\varphi '} {\hat{a}}_z \bigg ]e^{-jl\varphi } \Bigg ]d\Omega ' \end{aligned}$$

summation on *l* is independant of ($\nabla ' \times$) operator and $d\Omega '$ integration. ( $\nabla ' \times$) operates on ${e^{jl'\varphi '}}\hat{a_x}$, ${e^{jl'\varphi '}}\hat{a_y}$ and ${e^{jl'\varphi '}}\hat{a_z}$ which can for example be calculated as $\nabla ' \times ( { {e^{jl'\varphi '}}\hat{{a_z}}}) = \frac{{jl'}}{{\rho '}}{e^{jl'\varphi '}}{{\hat{a}}_{\rho '}}$ where ${\hat{a}}_{\rho '}$ will be replaced with equivalent Cartesian unit vectors. Eventually (17) takes the following form

18 $$\begin{aligned} {\textbf{H}}^\text {loc} - {\textbf{H}}^\text {ext} = \frac{{d}}{8j}{\alpha _m} \sum \limits _l \Bigg [ {\int}\sum \limits _{l'} { jl' e^{jl'\varphi '}} \bigg [ (b_{l'}^{z'}*{a}_{l,l'}) \left( \frac{\cos \varphi ' }{\rho '} \right)\hat{a_x} + (b_{l'}^{z'}*{{a}_{l,l'}}) \left( \frac{\sin \varphi ' }{\rho '} \right)\hat{a_y} \nonumber \\ -\left( ({b_{l'}^{x'}*{{a}_{l,l'}}}) \left( {\frac{{ \sin \varphi '}}{{\rho '}}} \right)+ ({b_{l'}^{y'}*{{a}_{l,l'}}}) \left(\frac{{\cos \varphi '}}{{\rho '}} \right)\right) {{\hat{a}}_z} \bigg ] d\Omega ' \Bigg ] {e^{-jl\varphi }} \end{aligned}$$

Due to the mentioned singularity in evaluating localized field, angular region of singularity $\Delta \varphi =\Delta P / nd$ where *nd* is the approximate radial distance of unit cell and *n* is in the order of 1000, will be subtracted from the integration region so $\left( {\prod {(\frac{{\varphi ' - 2\pi }}{{2\pi }}) - \prod {(\frac{{\varphi '}}{{\Delta \varphi }})} } } \right) \left\| {ds'} \right\| d\varphi ' dz$ replaces $d\Omega '$. Another Fourier series with $d_{l'}$ coefficient assumed as follows

19 $$\begin{aligned} \left[ {\prod {(\frac{{\varphi ' - 2\pi }}{{2\pi }}) - \prod {(\frac{{\varphi '}}{{\Delta \varphi }})} } } \right] \frac{\left\| {ds'} \right\| }{\rho ' }=\sum \limits _{l'} d_{l'}{e^{jl'\varphi '}} \end{aligned}$$

multiplying the mentioned Fourier series by the other items in (18) summarized to a single Fourier series where $d'_{l'}$ replaces ($\sin \varphi 'd_{l'}$) and $d''_{l'}$ replaces ($\cos \varphi 'd_{l'}$)

20 $$\begin{aligned} {\textbf{H}}^\text {loc} - {\textbf{H}}^\text {ext} = \frac{{d}}{8j}{\alpha _m} \sum \limits _l \Bigg [ {\int}\sum \limits _{l'} { jl' e^{jl'\varphi '}} \bigg [ (b_{l'}^{z'}*{a}_{l,l'}*d''_{l'})\hat{a_x} + (b_{l'}^{z'}*{a}_{l,l'}*d'_{l'}) \hat{a_y} \nonumber \\ -\left( ({b_{l'}^{x'}*{a}_{l,l'}}*d'_{l'}) + ({b_{l'}^{y'}*{a}_{l,l'}}*d''_{l'})\right) {{\hat{a}}_z} \bigg ] d\varphi ' dz \Bigg ] {e^{-jl\varphi }} \end{aligned}$$

($a_{l,l'}*d'_{l'}$) simplified to $a'_{l,l'}$ and ($a_{l,l'}*d''_{l'}$) to $a''_{l,l'}$. For the convolution operations we pursue the following approach^23^

21 $$\begin{aligned} \sum \limits _{l'} { jl' e^{jl'\varphi '}}(b_{l'}^{x'}*{a'}_{l,l'})=\sum \limits _{u'}\sum \limits _{l'}jl'(b_{l'}^{x'}{a'}_{l,u'-l'})e^{ju'\varphi '} \end{aligned}$$

which reduces all the convolutions to the multiplication of coefficients as follows

22 $$\begin{aligned} {\textbf{H}}^\text {loc} - {\textbf{H}}^\text {ext}= & {} \frac{d}{8j}{\alpha _m}\sum \limits _l \Bigg [ {\int} \sum \limits _{u'}\sum \limits _{l'} jl'\bigg [ (b_{l'}^{z'}a''_{l,u' - l'})\hat{a_x} + (b_{l'}^{z'}a'_{l,u'- l'})\hat{a_y}-(b_{l'}^{x'}a'_{l,u' - l'} + b_{l'}^{y'}a''_{l,u' - l'})\hat{a_z}\bigg ]\nonumber \\{} & {} e^{ju'\varphi '} d\varphi ' dz \Bigg ] e^{-jl\varphi } \end{aligned}$$

Now according to the Fourier series properties, following integral of $\varphi '$ in $2\pi$ interval reduces to a constant item of the corresponding series where $u'=0$ and we have

23 $$\begin{aligned} {\textbf{H}}^\text {loc} - {\textbf{H}}^\text {ext}= & {} \frac{{d}}{8j}{\alpha _m}\sum \limits _l \Bigg [ \sum \limits _{l'} jl' \bigg [(b_{l'}^{z'}a''_{l, -l'})\hat{a_x} + (b_{l'}^{z'}a'_{l,-l'})\hat{a_y}-(b_{l'}^{x'}a'_{l,- l'}+b_{l'}^{y'}a''_{l,- l'})\hat{a_z} \bigg ] \Bigg ] e^{-jl\varphi }\nonumber \\= & {} \frac{{d}}{8}{\alpha _m}\sum \limits _l \Bigg [ \sum \limits _{l'} l' \bigg [H_{l,l'}^{s,x}\hat{a_x} + H_{l,l'}^{s,y}\hat{a_y}+H_{l,l'}^{s,z}\hat{a_z} \bigg ] \Bigg ] e^{-jl\varphi } \end{aligned}$$

### Analysis verification

#### Excitation and surface reflectivity

The theory presented in previous section can be used to calculate the localized field on surfaces like $\rho$ in the presence of appropriate external field which leads to obtaining reflection coefficient on the surface.

Line source $I_0$ at $(\rho _0, \varphi _0)$ with $\omega =2\pi \times 2.67$ THz considered as an external source^24^ proved that this source produces a $TM^z$ wave in $\rho \ll \rho _o$ with the following E-field

24 $$\begin{aligned} E_z^{\text{ext}} = \frac{{ - \omega \mu _0 I_0}}{4}H_{0}^2(k\left| {\mathbf {\rho }- \mathbf {\rho }_0} \right| ) \end{aligned}$$

which is in the form of (8) and can be expanded in the Fourier series, Hankel part of $E^\text {ext}_z$ replaced by a Fourier series with ${f}_{l}$ coefficient

25 $$\begin{aligned} E_z^\text {ext} = \frac{{ - \omega \mu _0 I_0}}{4}\sum \limits _{l} f_{l}{e^{-jl\varphi }} \end{aligned}$$

and the corresponding transverse H-field components can be calculated as^24^

26 $$\begin{aligned} {\hat{a}}_z \times {\nabla _t} E^\text {ext}_z = j \omega {\mu _0} {{\textbf{H}}^\text {ext}_t} \end{aligned}$$

which results

27 $$\begin{aligned} {\hat{a}}_z \times \bigg [ \frac{{ - \omega \mu _0 I_0}}{4}\sum \limits _{l} -jlf_{l} \left[ \frac{\cos \varphi }{\rho }{\hat{a}}_x + \frac{\sin \varphi }{\rho } {\hat{a}}_y \right] e^{-jl\varphi } \bigg ] = j \omega {\mu _0} {{\textbf{H}}^\text {ext}_t} \end{aligned}$$

by substituting $\frac{\cos \varphi }{\rho }$ by a series with $g'_l$ coefficient and $\frac{\sin \varphi }{\rho }$ by a series with $g''_l$, we have the *x* component of external H-field as

28 $$\begin{aligned} H_x^\text {ext} = \frac{I_0}{4} \left[ \sum \limits _{l}l(f_{l}*g''_l)e^{-jl\varphi }\right] = \frac{I_0}{4}\left[ \sum \limits _{l}l H_{l}^{\text{ext},x} e^{-jl\varphi }\right] \end{aligned}$$

**Figure 5:**
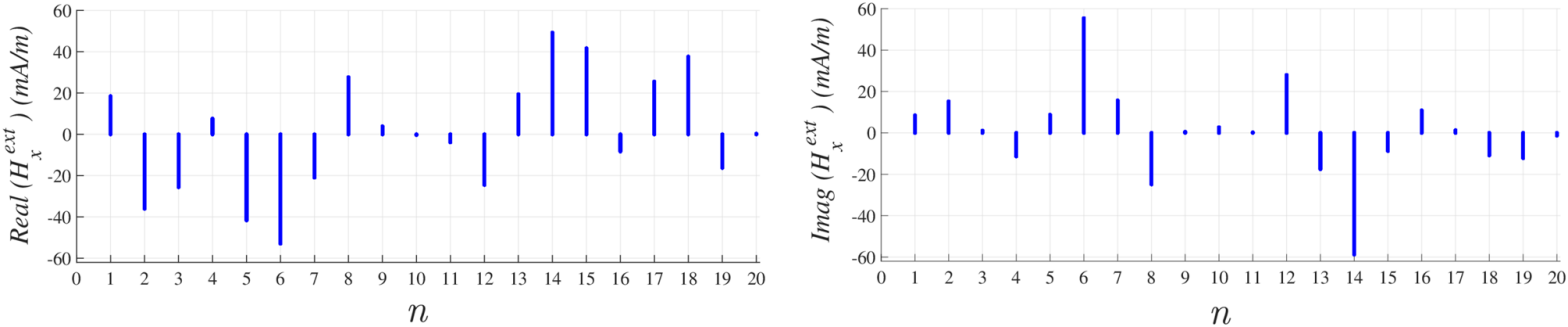
Real and imaginary part of evaluated Fourier series in Eq. (29) at each discreted $\varphi$ angle.

The normalized *x* component of external H-field is obtained as another Fourier series with $H_{l}^{\text {ext},x}$ coefficient

29 $$\begin{aligned} H_x^{\text {ext}} = \frac{1}{4}\sum \limits _{l}l H_{l}^{\text {ext},x} e^{-jl\varphi } \end{aligned}$$

which can be evaluated at 20 discrete values of $\varphi$ on $\rho$ surface where the surface was selected as an elliptical cylinder with cross-section equation $\rho = 2/ \sqrt{3 \cos ^2 \varphi +1}$. The evaluated Fourier series at each determined discrete value using MATLAB software demonstrated in Fig. 5.

**Figure 6:**
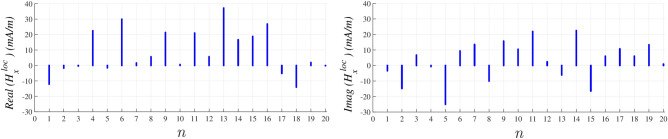
Real and imaginary part of evaluated Fourier series in Eq. (30) at each discreted $\varphi$ angle.

The overall *x* component of localized H-field in the presence of proposed external source is obtained using (11) as

30 $$\begin{aligned} H_x^\text {loc} = \frac{1}{4}\sum \limits _{l} \Big [ l H_{l}^{\text {ext},x} + \frac{{d}}{8}{\alpha _m} \sum \limits _{l'} l' H_{l,l'}^{\text {s},x}\Big ]e^{-jl\varphi } \end{aligned}$$

and demonstrated in Fig. 6 at the same 20 discrete values of $\varphi$ angle. Same procedure could be used to evaluate another series for $H_y^\text {loc}$ respectively, but the selected external source causes $H_y^\text {loc} \ll H_x^\text {loc}$ at $\rho \ll \rho _o$ and makes the *y* component of reflection coefficient less important.

**Figure 7:**
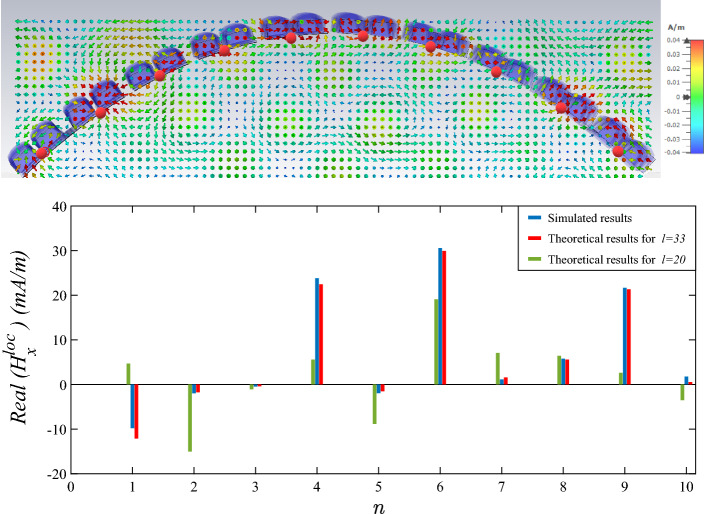
Real parts of distributed $H_x^\text {loc}$ at 10 discreted $\varphi$ angle which is compared to evaluated Fourier series for two different *l* in Eq.  (30).

On the one hand, it is obvious in Fig. 7 that the Fourier series coefficients number *l* in (30) converges to the obtained results for distributed $H_x^\text {loc}$ from CST Microwave Studio at $l=33$.

**Figure 8:**
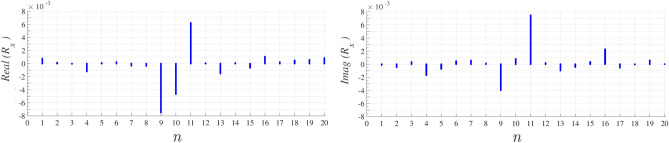
Real and imaginary part of evaluated $R_x$ at each discreted value of $\varphi$ angle.

After calculating localized wave, we need to achieve reflected wave from structure.The *x* component of reflected field $H_x^\text {ref}$ is calculated on each unit cell of proposed cylindrical surface with $a^2$ area according to^25^

31 $$\begin{aligned} H_x^{\text {ref}}= \frac{ - j\omega }{2\eta_0 a^2}{\alpha _m H_x^\text {loc}} \end{aligned}$$

hence the *x* component of reflection coefficient, $R_x$ is the ratio of obtained $H_x^{\text {ref}}$ and proposed $H_x^{\text {ext}}$ which is demonstrated in Fig. 8 at the same 20 discrete values of $\varphi$ angle. $Z_x$ can also be achieved directly from obtained $R_x$.

#### Graphene strip lines

**Figure 9:**
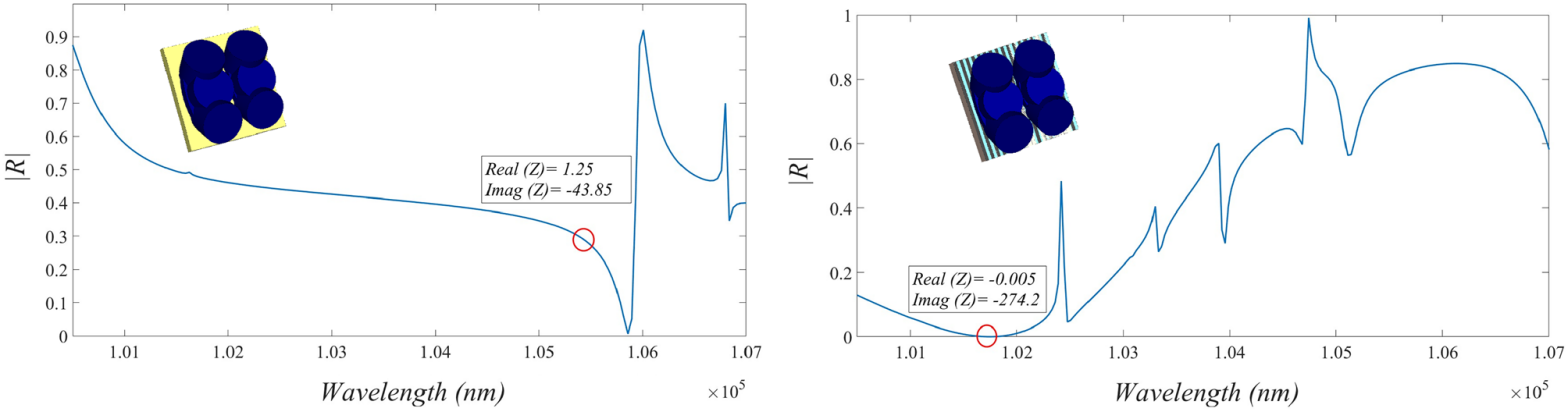
A unit cell reflectivity properties with applying graphene strip lines and without utilizing it.

Graphene Strip Lines are added as patch arrays to reduce the overall reflectivity of proposed metasurface which is examined for a unit cell in Fig. 9. It is clearly obvious that strip lines reduces overall reflectivity of our structure and eliminates the real part of impedance. Focusing on eliminating the imaginary part of impedance is beneficial in achieving the zero-reflection properties for the surface in the following. The impedance of this layer for TM-polarized incident field is determined as^26^

32 $$\begin{aligned} Z_{sl}^\text {TM} = j \alpha \frac{\eta _\text {eff}}{2} \left( 1 - \frac{k_0^2}{k_\text {eff}^2}\frac{\sin ^2\theta }{2}\right) \end{aligned}$$

where $k_0$ is the wave number in free space. Host medium incident wave number $k_\text {eff}$ and wave impedance of that $\eta _\text {eff}$ are obtained from relative effective permittivity ${\varepsilon _\text {eff}} = \frac{{\varepsilon _r} + 1}{2}$ where ${\varepsilon _r}$ in the desired graphene medium is defined as^27^

33 $$\begin{aligned} \varepsilon _r=\frac{1+j\sigma _g}{\omega \varepsilon _0 t_g} \end{aligned}$$

where the scalar surface conductivity of layer is $\sigma _g= \sigma _{\text {intra}}+\sigma _{\text {inter}}$ in which the former term is due to the intraband contributions and the latter term is due to the interband contributions. In mid-infrared frequencies where $\omega$ is below $2\mu _{c}/\hbar$, the interband term is negligible and the intraband term is dominant and equals^28^

34 $$\begin{aligned} \sigma _{\text {intra}}=j \frac{e^{2} k_B T}{\pi \hbar ^{2}(\omega -j 2 \Gamma )}\Bigg (\frac{\mu _{c}}{k_{B} T}+2 \ln \bigg ( exp({-\mu _{c}/k_{B}T}) +1\bigg )\Bigg ) \end{aligned}$$

where $\Gamma$ is a phenomenological scattering rate, $k_B$ is Boltzmann’s constant, $\hbar$ is the reduced Planck constant and *T* is the temperature.

$\theta$ in Eq. (32) is the angle of incident vector with $\rho$ surface which can be written in the terms of $\varphi$ angle as follows

35 $$\begin{aligned} \theta = {\cos ^{- 1}}\left[ \frac{ \rho (\varphi ) \cos \varphi + \frac{d\rho (\varphi )}{d\varphi } \sin \varphi }{\sqrt{\rho (\varphi )^2+1}} \right] \end{aligned}$$

and the grid parameter $\alpha$ in Eq. (32) is defined as^25^

36 $$\begin{aligned} \alpha = \frac{{k_\text {eff}D}}{\pi } \ln \bigg (\frac{1}{\sin \frac{\pi W}{2D}} \bigg ) \end{aligned}$$

where the strip width and their spacing are selected as $W=2.58$ $\mu m$, $D=5.16$ $\mu m$ for 2.67 THz frequency. It is crucial to note that every optimization involves making a trade-off. For instance, the use of strip lines can reduce reflectivity, but it also induces capacitive and inductive effects on the structure^29^, leading to undesired outcomes like frequency shifts in the main frequency.

**Figure 10:**
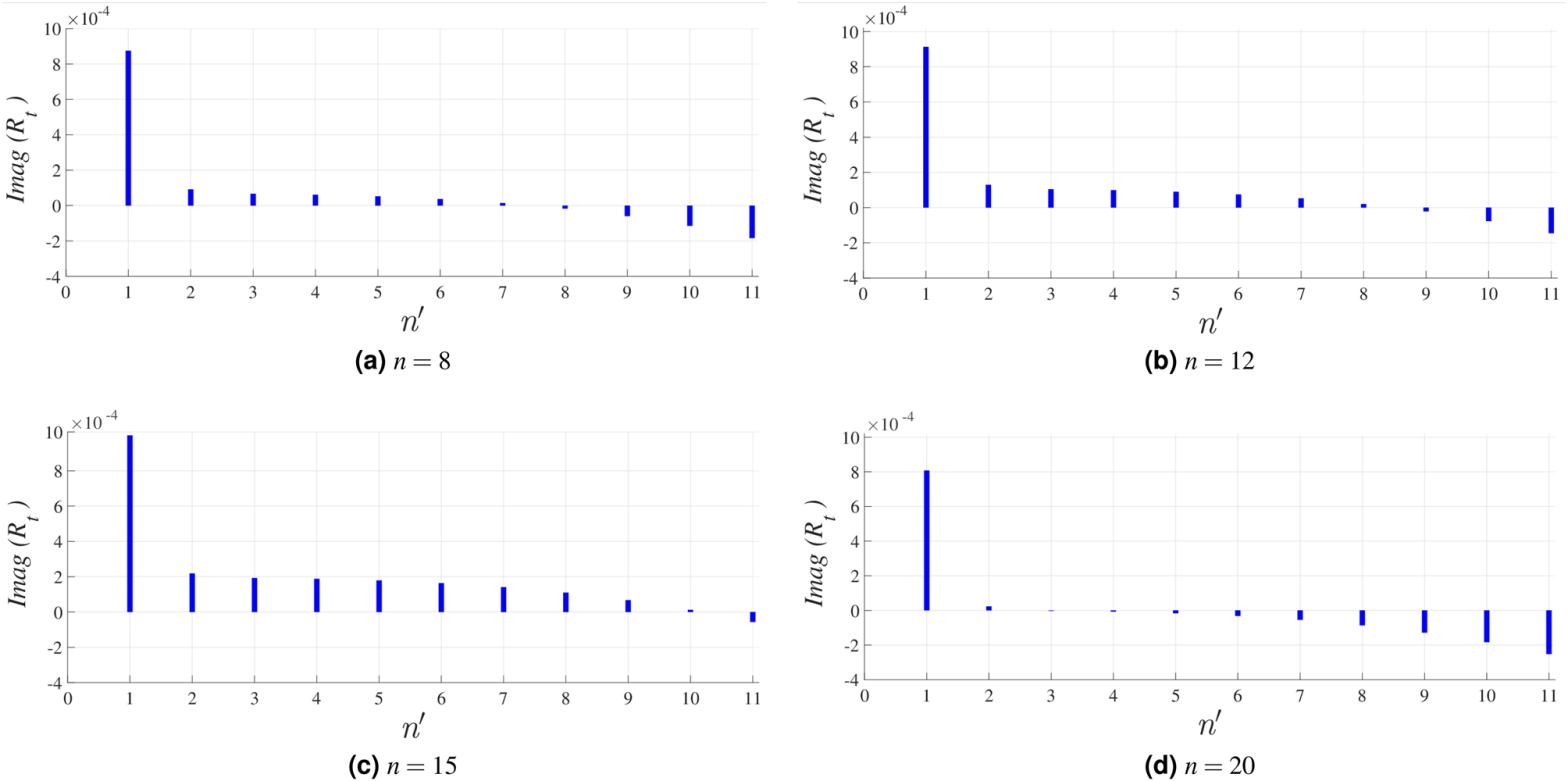
The imaginary part of $R_t$ in 4 different intervals of $\varphi$ angle.

#### Graphene ribbons

In this section, we employ a knock-out technique to achieve our ultimate goal of zero-reflection. By applying different potentials to graphene ribbons, we can attain the desired surface impedance, which can be manipulated to achieve zero-reflection properties.

**Table 1:** Optimized graphene chemical potential ($n^{\prime }$) selections.

*n*	1	2	3	4	5	6	7	8	9	10	11	12	13	14	15	16	17	18	19	20
$n^\prime$	2	2	10	1	2	11	11	7	1	11	11	8	1	2	10	11	2	2	11	3

Graphene ribbons with $t_g=10$ *nm* thickness and distinct values of chemical potential $\mu _{c}$ are added to each interval of $\varphi$ angle, *n*. $Z_g$ is caused by this layer

37 $$\begin{aligned} {Z_g} = \frac{{{t_g}}}{{{\sigma _g}}} \end{aligned}$$

**Figure 11:**
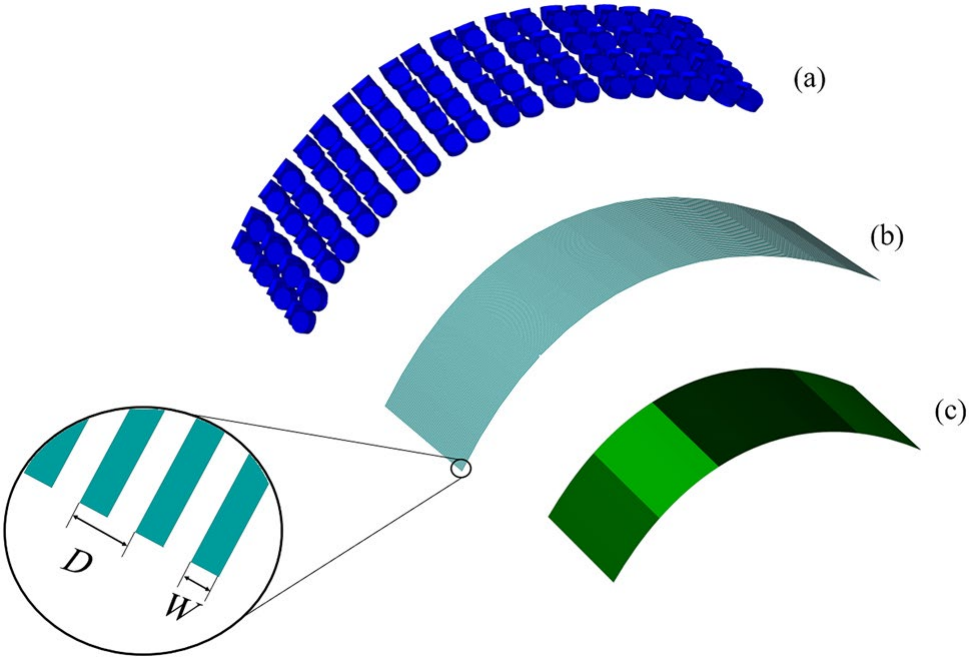
Proposed 3-layer hybrid structure.

**Figure 12:**
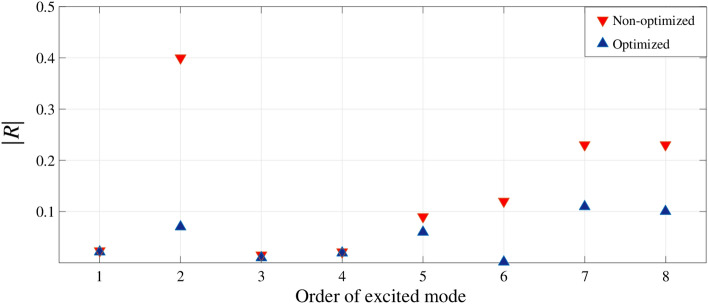
Reflection in each excited mode.

The overall total impedance of designed 3-layer hybrid structure is^30^

38 $$\begin{aligned} {Z_t} = \frac{1}{\frac{1}{Z_x} + \frac{1}{Z_g} + \frac{1}{Z^\text {TM}_{sl}}} \end{aligned}$$

the total reflectivity of the structure $R_t$, obtained from the overall total impedance, is dependant to $\mu _{c}$ of the selected ribbons in each interval of $\varphi$ angle, *n*. The selection procedure will done by observing the imaginary part of $R_t$ in the presence of 11 different quantities of $\mu _c$ in [0, 1] interval of it, denoted by $n^\prime$. The imaginary part of conductivity plays an important role in the propagation of surface waves guided by the graphene sheet^31^ so the optimizing procedure is restricted to cancelling the imaginary part of $R_t$. Figure 10 shows the imaginary part of $R_t$ in 4 different intervals of $\varphi$ angle, *n* for 11 possible selections of $\mu _c$, $n^\prime$. All the optimized $\mu _c$ selections are shown in Table 1.

Finally a reasonable segment of the optimized 3-layer hybrid structure involving five intervals of $\varphi$ angle from $n=8$ to $n=12$ as Fig. 11 is simulated in CST Microwave Studio to validate the accomplished optimization procedure. In non-optimized case all the graphene ribbons are simulated with $\mu _c=0$. Excited modes are obtained due to the Eigenmode Solver for both optimized and non-optimized structures and the reflectivity |*R*| in corresponding wavelengths are compared in each mode as Fig. 12.

**Figure 13:**
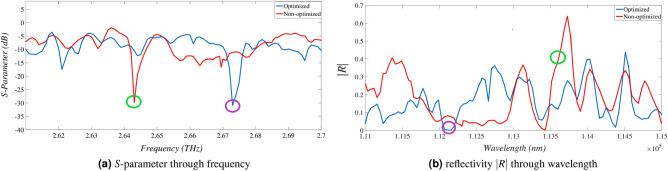
Results for 2nd excited mode where optimization details are marked.

By applying different chemical potentials to graphene ribbons and achieving varying conductivities, excitation modes may be formed with slight shifts in their frequencies. For instance, the second mode of the non-optimized structure appears at 2.64 THz with an equivalent reflectivity of approximately 0.4, while the second mode of the optimized structure appears at 2.67 THz with an equivalent reflectivity near to zero. The S-parameter and the reflectivity |*R*| are showed in details as Fig. 13.

## Discussion

In summary, we introduced a novel approach to determine the electromagnetic response of dielectric-graphene hybrid curved metasurfaces due to excitation of the toroidal moment. Analyzed metasurface with asymmetric unit cells designed for exciting the well-known trapped mode whereas they show a robust response for oblique incidents. Trapped mode excitation and the concept of bound states in the continuum (BIC) do not merely provide the high quality properties for proposed curved structure and accentuates our introduced novel approach for determining localized electromagnetic fields. Consequently, any practical surface with random and arbitrary curve can be optimized by utilizing the near-field responses. To figure out the functionality of the procedure, a reasonable segment of the designed Silicon metasurface is optimized using graphene ribbons and strip lines which results near-zero reflectivity properties for the structure. To bias graphene sheets empirically, one can apply an external voltage to the sheets using a voltage source or bias tee. Graphene can be indirectly stimulated by applying thin gold electrodes on the side borders of the structure and mounting the graphene layers onto them. By applying stimulation to the metal electrodes, the graphene layers can be activated or manipulated to achieve the desired conductivity or impedance properties. It is important to note that the exact procedure for mounting and stimulating graphene layers may depend on the specific experimental setup and conditions, and may require careful calibration and optimization to achieve the desired results^32^. In a more advanced approach, tension-sensitive resistors can be utilized in a way that allows the change of curvature to result in a variable voltage. This can enable the structure to self-adjust, as the resistors can dynamically respond to changes in the curvature or deformation of the structure.

## Data Availability

The datasets used and/or analysed during the current study available from the corresponding author on reasonable request.

## References

[1] Khatami MS, Dehmollaian M, Yousefi L (2021). Analysis of wave scattering from 2d curved metasurfaces using floquet and fourier series expansions. IET Microwaves Antennas Propag..

[2] Johnsen S (2001). Hidden in plain sight: The ecology and physiology of organismal transparency. Biol. Bull..

[3] Binetti VR, Schiffman JD, Leaffer OD, Spanier JE, Schauer CL (2009). The natural transparency and piezoelectric response of the greta oto butterfly wing. Integr. Biol..

[4] Siddique RH, Gomard G, Hölscher H (2015). The role of random nanostructures for the omnidirectional anti-reflection properties of the glasswing butterfly. Nat. Commun..

[5] Babicheva VE, Evlyukhin AB (2018). Metasurfaces with electric quadrupole and magnetic dipole resonant coupling. ACS Photon..

[6] Dubovik V, Tugushev V (1990). Toroid moments in electrodynamics and solid-state physics. Phys. Rep..

[7] Kaelberer T, Fedotov VA, Papasimakis N, Tsai DP, Zheludev NI (2010). Toroidal dipolar response in a metamaterial. Science.

[8] Dong Z-G, Ni P, Zhu J, Yin X, Zhang X (2012). Toroidal dipole response in a multifold double-ring metamaterial. Opt. Exp..

[9] Tuz VR, Khardikov VV, Kivshar YS (2018). All-dielectric resonant metasurfaces with a strong toroidal response. ACS Photon..

[10] Basharin AA (2015). Dielectric metamaterials with toroidal dipolar response. Phys. Rev. X.

[11] García-Etxarri A (2011). Strong magnetic response of submicron silicon particles in the infrared. Opt. Exp..

[12] Fedotov V, Rose M, Prosvirnin S, Papasimakis N, Zheludev N (2007). Sharp trapped-mode resonances in planar metamaterials with a broken structural symmetry. Phys. Rev. Lett..

[13] Hsu CW, Zhen B, Stone AD, Joannopoulos JD, Soljačić M (2016). Bound states in the continuum. Nat. Rev. Mater..

[14] He Y, Guo G, Feng T, Xu Y, Miroshnichenko AE (2018). Toroidal dipole bound states in the continuum. Phys. Rev. B.

[15] Gupta M, Srivastava YK, Manjappa M, Singh R (2017). Sensing with toroidal metamaterial. Appl. Phys. Lett..

[16] Srivastava YK (2019). Terahertz sensing of 7 nm dielectric film with bound states in the continuum metasurfaces. Appl. Phys. Lett..

[17] Ederer C, Spaldin NA (2007). Towards a microscopic theory of toroidal moments in bulk periodic crystals. Phys. Rev. B.

[18] He Y, Guo G, Feng T, Xu Y, Miroshnichenko AE (2018). Toroidal dipole bound states in the continuum. Phys. Rev. B.

[19] Miroshnichenko AE (2015). Nonradiating anapole modes in dielectric nanoparticles. Nat. Commun..

[20] Chew, W. C. Dyadic Green’s Functions, 375–428 (1995).

[21] Cohen LD, Haracz RD, Cohen A, Acquista C (1983). Scattering of light from arbitrarily oriented finite cylinders. Appl. Opt..

[22] Palik ED (1998). Handbook of optical constants of solids.

[23] Mota, F. Fourier series and transforms via convolution. arXiv preprint arXiv:2201.03974 (2022).

[24] Dudley, D. G. Electromagnetic Boundary Value Problems, 181–245 (1994).

[25] Tretyakov, S. Analytical modeling in applied electromagnetics (Artech House, 2003).

[26] Luukkonen O (2008). Simple and accurate analytical model of planar grids and high-impedance surfaces comprising metal strips or patches. IEEE Trans. Antennas Propag..

[27] Bellucci S (2018). Electrical permittivity and conductivity of a graphene nanoplatelet contact in the microwave range. Materials.

[28] Hanson GW (2008). Dyadic green’s functions and guided surface waves for a surface conductivity model of graphene. J. Appl. Phys..

[29] Gao X (2018). A reconfigurable broadband polarization converter based on an active metasurface. IEEE Trans. Antennas Propag..

[30] Kim M, Eleftheriades GV (2018). Design and demonstration of impedance-matched dual-band chiral metasurfaces. Sci. Rep..

[31] Mikhailov SA, Ziegler K (2007). New electromagnetic mode in graphene. Phys. Rev. Lett..

[32] Rezaei MH, Shiri M (2021). High-performance tunable resonant electro-optical modulator based on suspended graphene waveguides. Opt. Express.

